# Digital intraoral scanner devices: a validation study based on common evaluation criteria

**DOI:** 10.1186/s12903-022-02176-4

**Published:** 2022-04-26

**Authors:** Ivett Róth, Alexandra Czigola, Dóra Fehér, Viktória Vitai, Gellért Levente Joós-Kovács, Péter Hermann, Judit Borbély, Bálint Vecsei

**Affiliations:** Department of Prosthodontics, Semmelweis University, Szentkiralyi Street 47, 1088 Budapest, Hungary

**Keywords:** Digital impression-taking, Intraoral scanner, Validation

## Abstract

**Background:**

The evolution of intraoral scanners (IOSs) is rapid, and new IOSs appear on the market with different properties depending on the manufacturers. There is no uniform rating system based on a defined set of aspects that has reported in the literature that can be used to compare these devices. This validation study aimed to compare different IOSs based on objective and comprehensive parameters.

**Methods:**

In this study, 12 different IOSs were examined. The IOSs that were tested in this study in order of their delivery included the 3Shape Trios 3 Pod®, Planmeca Emerald®, Straumann DWIO®, GC Aadva®, iTero Element 2®, CEREC Primescan®, Medit i500®, 3Shape Trios 4 Move®, Carestream CS3600®, 3Shape Trios 4 Pod®, Carestream CS3700®, and Planmeca Emerald S®. IOSs were evaluated in four different ways: (a)summary chart, (b)comparative assessment, (c)data based on in vitro measurements and (d)accuracy measurements. A scoring system was created to enable an objective rating of IOSs.

**Results:**

The differences among IOSs were demonstrated in point scores (summary chart[max. 10 points] + weight of IOSs[max. 2.5 points] + circumference of IOSs[max. 2.5 points] + in vitro scanning time[max. 2.5 points] + pauses in data capture[max. 2.5 points] + accuracy[max. 10 points] = summary[max. 30 points]). Trios 4 Pod achieved the greatest cumulative score (23.37 points), furthermore it earned the highest points for summary chart and scanning speed. Regarding scanning continuity, the best-performing IOSs, which tied at identical point scores, were the Trios 3 and 4 Pod, Trios 4 Move, iTero Element 2, CS3600 and CS3700. The most accurate IOS was the CEREC Primescan, although it earned the lowest points of the comparative assessment (heaviest IOS). GC Aadva scored 5.73 points of a maximum of 30 points, which was the poorest result in this study.

**Conclusion:**

The scoring system reflects the differences among IOS devices based on the evaluated objective parameters and can be used to help clinicians select the right IOS device. The new generations of IOSs have more special properties, and their accuracy is higher than the previous versions.

*Trial registration* The permission for this study was granted by University Ethics Committee of Semmelweis University (SE RKEB number:108/2019).

## Background

The widespread use of computer-aided design/computer-aided manufacturing (CAD/CAM) technology in dentistry poses new challenges and goals for dentists. The integration of intraoral scanning systems into the digital dental workflow creates new solutions for dental treatment. There are two types of initial steps of CAD/CAM technology: direct and indirect imaging method. During indirect approach the workflow starts with a traditional impression taking, then the stone cast is scanned by a laboratory scanner [1]. The first step of the direct CAD/CAM workflow is to take an optical impression with intraoral scanner (IOS) devices [2, 3]. The accuracy of digital impression-taking required to ensure a successful clinical workflow has been demonstrated in scientific studies [2, 4–11]. Optical impressions made by IOSs have many advantages, and their implementation is important in clinical practice. Of note, the benefits of intraoral scanning include decreased gag reflex and working time, elimination of impression material or gypsum deformation, better communication with the laboratory, and easy repeatability [2, 8, 12]. Digital impression-taking also has some disadvantages: based on previous studies, a conventional impression might be a better option for addressing difficult prosthodontic situations (e.g., long-span restoration on multiple implants) [9, 10], and deep margin line detection or bite registration can be challenging. Other limitations of digital impression-taking are scanning fees to pay (with closed systems) and the costs of IOS devices, which are still expensive [3]. IOS devices are a way for dentists to partake in a digital workflow; however, a certain degree of experience is required to operate an IOS [13]. Hence, IOSs have a learning curve; thus, the members of the dental team need to spend time in training to effectively apply these devices in clinical practice [3, 14]. Dr. Francois Duret demonstrated the first CAD/CAM system at the France Dental Association's International Congress in 1985. The first application of digital impression-taking and chairside milling procedures in practice was in 1989 at the Midwinter Meeting, where Dr. Duret created a crown for a real patient in four hours [15, 16]. Of note, in 1987, when CEREC 1 (Siemens Corporation) was released, it was a chairside CAD/CAM system developed for inlays and onlays [17]. The second generation of CEREC (CEREC 2) was introduced in 1994 and could be used for making crowns. The third generation, CEREC 3, was introduced by Sirona Dental Systems, and dentists worldwide currently use this generation for making inlays, onlays, crowns and short bridges. The CEREC system has improved over the years, and 3 generations of CEREC IOSs have been developed: CEREC Bluecam/Omnicam/Primescan. The latest version, the CEREC Primescan IOS, can also be used for full-arch scans and chairside applications [18, 19]. Since then, several IOSs have been introduced to the dental market with different properties depending on manufacturers. There are some reviews about IOS efficiency, accuracy, speed, and learning curves [2, 3, 20–22]. There is no uniform rating system in the literature to compare these devices based on a defined set of aspects. On behalf of long-term dental application, a validation study based on the same objective parameters to evaluate IOSs was needed. Such a study could be essential for a clinician buying a new IOS. Traditional impression-taking techniques are the most common methods used in an average clinic in Hungary, but the evolution of new processes is rapid. Clinicians are beginning to invest in IOSs, and this progress has seemed to accelerate recently. The main challenge faced by universities is implementing these up-to-date skills. Pertinently, dental students have advantages in this regard because their skills in digital impression-taking methods are the same as those of clinicians [13]. Dental medical students have another advantage: openness to innovative digital technologies. Notably, awareness of the differences among IOSs is essential for long-term development [2]. This validation study aimed to compare different IOS devices based on objective and comprehensive parameters.

Permission for this study was granted by the University Ethics Committee of Semmelweis University (SE RKEB number: 108/2019).

## Methods

### Intraoral scanners

In this study, 12 different IOSs from 8 different manufacturers were examined. The 12 tested IOSs are listed in Table 1. Seven of these IOSs were pod versions (3Shape Trios 3 Pod®, Planmeca Emerald®, Medit i500®, CS3600®, 3Shape Trios 4 Pod, CS3700®, and Planmeca Emerald S®) that are connectable to high-performance laptops provided by Hungarian distributors. Straumann DWIO® and 3Shape Trios 4 Move® were portable IOSs, and the other IOSs (GC Aadva®, iTero Element 2®, CEREC Primescan®) were cart version devices with built-in computers. At the time of every experiment each examined intraoral scanner were primarily used and had the newest hardware versions available on the Hungarian market and every distributor provided the latest software versions of each intraoral scanner on the best performance computers recommended by the manufacturer [23]. Each IOS spent two weeks at the Department of Prosthodontics while the measurements were being performed.

**Table 1:** The 12 tested IOSs in order of delivery

Intraoral scanner	Place of manufacture	Year of manufacture
3Shape Trios 3 Pod®	Copenhagen, Denmark	2015
Planmeca Emerald®	Helsinki, Finland	2017
Straumann DWIO®	Basel, Switzerland	2015
GC Aadva®	Leuven, Belgium	2017
iTero Element 2®	Amsterdam, Netherlands	2018
CEREC Primescan®	York, PA, U.S	2019
Medit i500®	Seoul, South Korea	2018
3Shape Trios 4 Move®	Copenhagen, Denmark	2019
Carestream CS3600®	Atlanta, GA, U.S	2016
3Shape Trios 4 Pod®	Copenhagen, Denmark	2019
Carestream CS3700®	Atlanta, GA, U.S	2020
Planmeca Emerald S®	Helsinki, Finland	2020

### Participants of the study

Three dental students took part in each IOS testing procedure as operators (altogether 36 dental students were involved in tests). Dental students in their 6th or 10th  emester took part in this study. The students represented average students completing graduate education at Semmelweis University with no experience in intraoral scanning. They attended a presentation about the digital workflow and a demonstration about the use of the actual IOS before the measurements [24]. The lecture was held by a well-trained dentist, dental technician, or distributor who was an expert in the use of the actual IOS. In all cases, education was provided by the distributor company. During scanning, supervision was granted by a dentist (supervisor) with experience in digital impression-taking. It is known that the calibration of the intraoral scanners can have significant impact to their accuracy [25]. To exclude the distortion factor of the decalibration (caused by transportation, assembly, disassembly, scanning etc.) calibrations were performed on each measured intraoral scanner according to the manufacturer’s recommendations. First time, intraoral scanners were calibrated by the distributor after their arrival to the Department of Prosthodontics to show the proper method. Then calibrations were being performed by the study group to learn the procedure. Calibrations were done by each operator before and between model and clinical scannings. The operators were involved in in vivo and in vitro intraoral scanning. The whole data collection procedure was performed from June 2018 to December 2020.

### Evaluation of IOSs

The evaluation of the IOSs was performed in four different ways. These included (a) summary chart, (b) comparative assessment (weight of the IOS handpiece and the circumference of its head), (c) data based on in vitro measurements (scanning time and continuity of scanning process), and (d) accuracy measurements. The examined IOSs were rated based on these four components. A summary chart was made for each scanner. To determine the ergonomic design of the IOSs, the weight of the IOS handpiece and the circumference of its head were measured. During the measurements, a standard protocol was created to record the in vitro scanning time and continuity of the scanning process (number of pauses during data capture). Accuracy (trueness and precision) was also assessed from these in vitro data. The score points of the intraoral scanners were measured based on a dynamic scoring system. In vivo digital impressions were taken for evaluating the ergonomic properties of the intraoral scanners. The scores for the different parameters were determined based on their consideration in terms of use. The two most important aspects of buying a new intraoral scanner device are the accuracy of the devices and the special properties of the intraoral scanners [26]. Furthermore, the most of examined parameters were incorporated in summary chart and accuracy measurements. In our summary chart 26 special properties of the intraoral scanner devices were listed and during accuracy measurements 5 different parameters were examined and evaluated. Accordingly, in our research the summary chart and the accuracy measurements worth the highest points (10–10 points). The scanning experience of the operators could influence the scanning speed and continuity of scanning process, these parameters do not depend only on the device [2]. In addition, the ergonomic properties of the intraoral scanners mostly affect the comfort of intraoral scanners’ usage. Therefore, these parameters worth 2,5 points in our scoring system.

### Summary chart

In our summary chart, 26 properties were listed (Table 2), including the features described below.

1. Hardware specifications:
  - *Color:* All the tested intraoral scanners in our study produced color impressions. Regarding color, two types were distinguished—realistic (1 point) and unrealistic (0.5 points) color. The high-quality digital impression in lifelike colors means that the operator can analyze and follow the chronological changes of the patient’s oral cavity based on virtual model (e.g., abrasion, caries, gum recession) [2]. Some intraoral scanners can make colorful digital impressions but use unrealistic colors for digitizing the oral cavity. These digital impressions are not suitable for realize or follow any lesions in oral cavity. The unrealistic colored virtual models have less information than the realistic colored digital impressions.
  - *Touchscreen:* Indicated whether the examined IOS had a touchscreen (1 point) or not (zero points).
  - *Remote controller:* Denoted whether the examined IOS had a remote controller (1 point) or not (zero points).
2. Configuration: The design of the IOS was also evaluated: 0 points were awarded if an IOS was available in a single configuration only, 1 point was awarded if there was more than one configuration (e.g., pod version/portable IOS/cart versions).
3. Exportable file type: Sending files is more comfortable and faster if an IOS has its own cloud-based platform; such IOSs were awarded 1 point in our summary chart.
4. Open vs. closed system: Scanning systems can differ based on the possibility of whether there is a free interface with all available CAD software (open versus closed systems) [2]. In case of open systems (1 point), intraoral scanners permit the direct export of the mainly used STL files (or PLY files) therefore 3D files could be sent to any type of CAD software [26]; however, in closed systems (zero points) the intraoral scan could be forwarded only to the manufacturer’s CAD system.
5. Chairside: If the examined IOS had its own chairside system, it was awarded 1 point.
6. Application: The presence of an implantology or orthodontics system each earned the examined IOS 1 point.
7. Special properties: Each special property earned 1 point; these properties included tooth shade and prepared tooth shade selection support, individual jaw movement detection, prepreparation/post and core/impressions scan support, denture workflow, smile design/caries detection feature, and variable scanning tip size. Three more properties were listed without naming them (“other special property”) if an IOS had features that were not listed above.
8. License of the software: The examined IOS was awarded zero points if an annual license was the only available option (this means the user must pay a license fee every year to use the IOS) and the IOS was awarded 1 point if it had a perpetual license option.
9. Infrastructure: The infrastructure consists of two aspects: customer service and availability of training. Service: zero points for “no service”, 0.5 points for foreign or online service and 1 point for the presence of a domestic service center. Training: 0 points if there is no training available from the distributor, 0.5 points for foreign or online coaching and 1 point for domestic training.

**Table 2:** Summary chart based on 26 properties

Hardware	Color	Not realistic: 0,5	Realistic: 1
	Touchscreen	No: 0	Yes: 1
	Remote controller	No: 0	Yes: 1
Configuration	Available in one configuration: 0		
	More than one configuration available: 1		
Exportable file type	Proprietary cloud-based platform: 1		
Open vs. closed system	Closed: 0		
	Open: 1		
Chairside	Proprietary chairside system: 1		
Application	Implantology: 1		
	Orthodontics: 1		
Specialized properties	Tooth shade selection: 1		
	Prepared tooth shade selection: 1		
	Individual jaw movement detection: 1		
	Prepreparation scan: 1		
	Post and core scan: 1		
	Impression scan: 1		
	Denture workflow: 1		
	Different sizes of scanning tips: 1		
	Smile design: 1		
	Caries detection: 1		
	Other special property: 1		
	Other special property: 1		
	Other special property: 1		
Software license	annual license: 0		
	perpetual license: 1		
Infrastructure	Service	Training opportunity	
	No service: 0 Foreign/online service: 0,5 Domestic service: 1	None provided: 0 Foreign/online: 0,5 Domestic: 1	

For every IOS, we evaluated how many properties were inherent for the device, and these features were converted into points based on a dynamic scoring system. The summary chart was made by an experienced dentist based on the literature, information given by the distributor company and our own experience with the IOS.

### Ergonomic design

To determine the ergonomic design of the IOSs, the circumference of the head of the IOS and the weight of the handpiece were measured. The circumference of the IOS head was measured by a tape measure three times, and then the average of the results was recorded. The same weighing scale was used for measuring the weight of the IOS handpieces three times. The three measurements, for both the head and the handpiece weight, were averaged, and both results were recorded. A dynamic scoring system was used to compare the IOSs to each other. The measurements were made by an experienced dentist.

### In vitro research

**Fig. 1:**
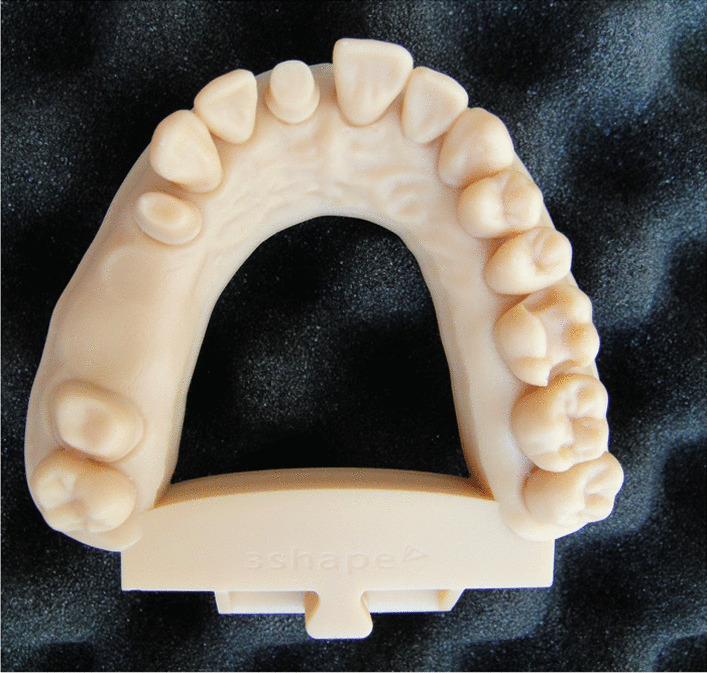
A polymethyl-methacrylate (PMMA) maxillary model that was used as a reference

**Fig. 2:**
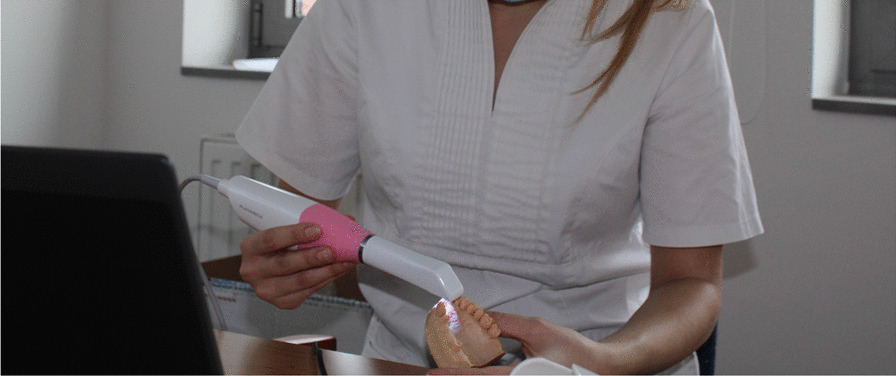
The reference cast was scanned by three undergraduate dental students who had no previous experience in intraoral scanning

**Fig. 3:**
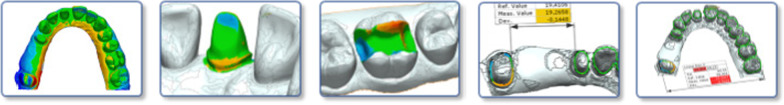
The five different parameters measured for the accuracy assessment of the intraoral scanners

In vitro measurements have the dual purpose of evaluating scanning time and continuity as well as measuring scanning accuracy. A polymethyl-methacrylate (PMMA) maxillary model was used as a reference (Fig. 1). Prepared teeth (FDI World Dental Federation) included numbers 11, 14, 17 for a crown and 26 for an inlay; teeth 15 and 16 were missing. A highly accurate industrial precision scanner (stereoSCAN neo; AICON 3D Systems GmbH, Braunschweig, Germany) was used to generate a reference STL file from the maxillary PMMA model described previously. According to the user’s guide of stereoSCAN neo, the accuracy of the reference scanner has a maximal 8 µm feature accuracy (and a 2-µm resolution limit) [27]. The reference cast was scanned by three undergraduate dental students who had no previous experience in intraoral scanning (Fig. 2). The three students scanned teeth ten times with an IOS. During scanning, students were supervised by an experienced dentist. According to the scanning strategy and IOS settings, the manufacturer’s instructions were followed, which were presented during the previous education period. When the manufacturer did not offer an ideal scanning strategy, the path started from the occlusal-palatal surface of the last right molar, holding the head of the IOS at a 45° angle, alongside the jaw, and then back on the buccal-occlusal surface [28]. Scanning times of and the pauses (continuity) during the data capturing process were also registered at each measurement, and then the averages for both aspects were calculated. The IOSs were also scored according to the average number of stops during scanning. All model scanning ended with the exportation of data in STL format. In the end, 30 STL files were created. The 30 STL files were exported, and 15 were randomly selected (5–5-5 data from each operator) for the inspection procedure. Geomagic Verify (v2015.2.0; 3D Systems, 333 Three D Systems Circle, Rock Hill, USA) inspection software was used to process the digital files and obtain the accuracy values of the IOSs. Accuracy was measured based on a previous study performed by Vecsei et al. [11]. The digital models were cut and trimmed to remove unnecessary parts to make the arches uniform. Then, the digital models were cropped to obtain the desired data for the different measurements: the prepared incisor, the inlay cavity with the adjacent tooth's approximal surfaces, and the four-unit bridge's area were digitally separated from the model and exported as STL files for the inspection process. One operator made all the STL file modifications. During the inspection process, we used the best-fit algorithm of the program to match each scan file with the reference STL dataset. The deviations were measured on the entire dental arch and separately in the inlay cavity and on the prepared incisor on the superimposed data. For digital caliper measurements, an intersecting plane in the equator level of the first premolars was defined on the reference STL file to make reference points. The shortest distance between the four-unit bridge's abutments and the dental arch's distal range (distance between second molars' distobuccal cusps) were measured in the plane. The root mean square (RMS) of the deviation of surface points was exported into statistical software to evaluate the level of trueness. Statistical analyses were performed with IBM SPSS Statistics statistical software (v27.0; IBM Corp.). The normality of the data distribution was examined using the Kolmogorov–Smirnov test (StataCorp. Stata Statistical Software: Release 15. College Station, Texas: StataCorp LLC). In terms of accuracy, trueness and precision, digital scans were assessed based on ISO standard 5725 [29–31]. Importantly, trueness indicates the closeness of the arithmetic means of measured values to the real value of the scanned object. Precision indicates the agreement of the tested values. In this study, each IOS could potentially have a maximum of 10 points for accuracy (5 for trueness and 5 for precision maximum). The average of the 15 measurements was used to calculate the point scores for trueness and precision, and then these scores were summed to indicate the accuracy of the IOS. For a complete overview of accuracy, we measured five different parameters indicative of the accuracy of the IOSs (Fig. 3):

- The average deviation of the full-arch scan from the reference dataset reveals global accuracy.
- The surface of the prepared incisor is the smallest area we measured, indicating the best accuracy of the IOS – most manufacturers use this value to describe IOS accuracy.
- The average surface deviation from the reference for a segment of the inlay cavity and the adjacent approximal surfaces can imply accuracy for chairside systems.
- The distance between the abutment teeth for the four-unit bridge shows the distortion effect of an edentulous ridge.
- The distance between the second molars’ distobuccal cusps in a reference plane indicates the global distortion of the scanning accuracy of the full arch, which shows the appropriateness of the stitching method.

**Fig. 4:**
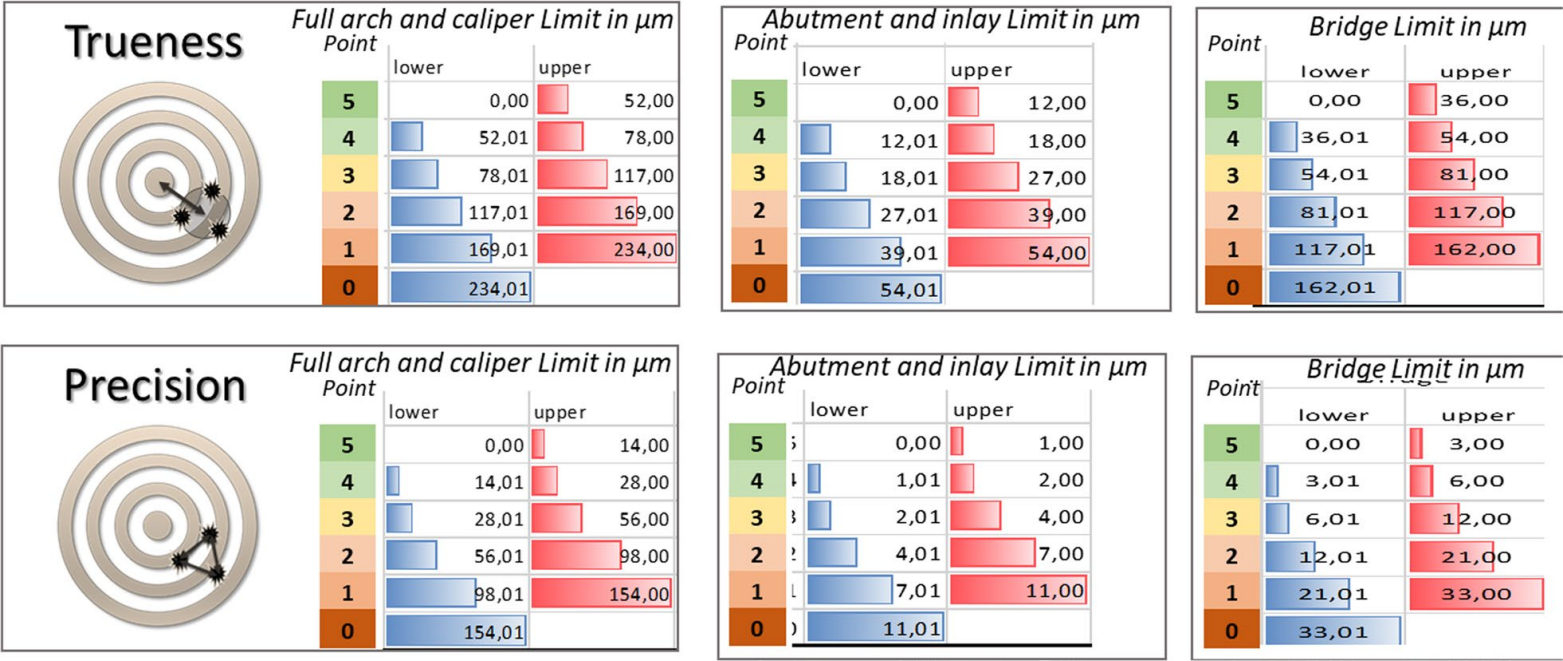
Ranges of limits and converted points based on area size

We established a scale to assign score points (grades) based on accuracy. The smaller the discrepancy was, the greater the score that the IOSs received. IOSs that were closer to the clinically unacceptable range received fewer points. The range and the converted points are listed based on the size of the area we checked (Fig. 4).

The acceptable tolerance was set at 54 µm ± 11 µm (trueness ± precision) for single-unit restorations (crown and inlay/onlay), 162 µm ± 33 µm for 4-unit restorations (little bridge) and 234 µm ± 154 µm for checking a full-arch scan. The range between zero and the tolerance value was separated into five ascending subranges. Each successive range was one and a half times the previous range. The acceptable value for the bridge was three times greater than that for a single-unit restoration. The averaged trueness and precision values in micrometers and the derived accuracy points are represented in Table 3.

**Table 3:**
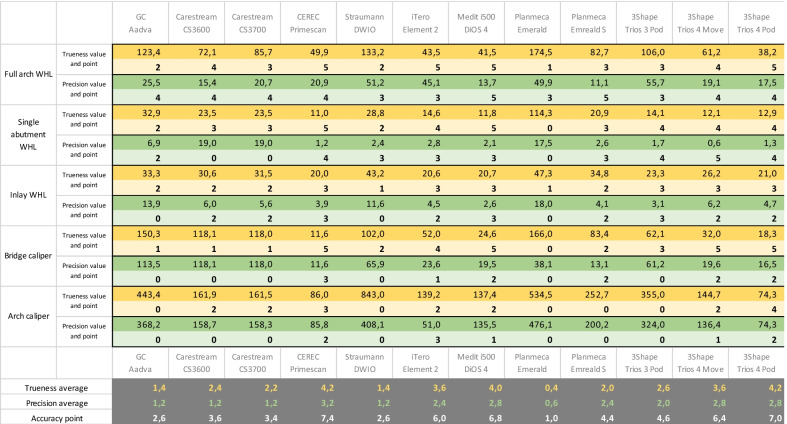
The averaged trueness and precision values in micrometers and the derived accuracy points

### In vivo research

In vivo scans were also made by the three dental students. Eighteen digital impressions were taken with each IOS (6 per student): 3 full-arch scans and 3 quadrant digital impressions from patients. The scans were performed at the same time after the in vitro scanning procedures. During scanning, supervision was granted by a dentist (supervisor) with experience in digital impression-taking. The inclusion criteria for patients included age over eighteen years, full dentition (except for the third molar), good oral hygiene, healthy hard/soft tissue (no caries or extraction socket), and normocclusion (Angle I). The exclusion criteria were orthodontic treatment in progress, dental implants, any prosthetic treatment (inlays/onlays, crowns or bridges), and periodontal disorders (gingivitis or periodontitis) [14].

Scanning procedures were performed according to the manufacturer’s instructions. If the system required it, the scanning device was calibrated before impression-taking began. Patient data and a digital order form were completed in each scanning case. If the scanning software supported the option of diagnostic scanning, this function was used for impression-taking. For better viewing, a retractor was applied (Optragate, Ivoclar Vivadent). A stopwatch was used to measure the scanning time. Total impression-taking time included scanning time required to take a complete impression of the upper and lower arches, to complete bite registration on the right and left sides, and to process and save the file. The total scanning time was measured from the commencement of patient data recording to the end of the processing procedure.

Full-arch study impressions were taken from the upper and lower arches by the examined IOS with bite registration in the intercuspidal position. If instructions were not given by the manufacturer, the same scanning strategy as described for the in vitro measurements was used. During quadrant scanning, students took digital impressions of the right upper and lower arches from the second molar to the canine. The last step was bite registration on the right side. After scanning the full-arches and quadrants, the virtual cast was accepted if all tooth surfaces were completely mapped, no crack lines were found, and bite registration was successful. The scanning procedure was repeated if a crack line appeared or if any other damage was detected on the virtual cast. In cases of missing data, additional images were taken. The total scanning times of full arch and quadrant scans were recorded and summed. In vivo scanning times were summarized in a chart but were not included in the scoring system.

### Evaluation by points

A scoring system was created for the objective rating of the IOSs. Points assigned to the IOSs were measured based on a dynamic scoring system for the summary chart, comparative assessment, and scanning time/continuity of scanning. Accuracy scores were determined based on a different method detailed in the previous section (“In vitro research”). Positioned at the top of the dynamic scale (maximum score) was the best-performing IOS among those assessed (summary chart, comparative assessment, scanning time, and continuity). Located at the bottom of the scale (0 points) was the IOS found to perform the worst in our testing procedures. A ranking scale was divided proportionately between the highest and the lowest score values; the assessed IOSs were then categorized on this scale based on their results. Finally, each IOS could be compared objectively to the other IOSs based on their features. The dynamic scale was subject to change if the currently assessed IOS performed better or worse than all other IOSs assessed thus far. In the scoring system, an IOS earned a potential maximum of 30 points.

The scoring system had three major components:

- Scoring based on the summary chart (maximum of 10 points)

In our summary chart, 26 properties are listed. For each IOS, its inherent properties were evaluated, and these features were converted into a scoring system with a dynamic scale. The top of the dynamic scale (maximum of 10 points) represented the best-performing IOS based on its properties. The bottom of the scale (0 points) represented the IOS with the worst properties in the summary chart.

b. Ergonomic design: IOS head circumference and handpiece weight (2.5 ± 2.5 = maximum of 5 points)

The weight and circumference of three often-used handpieces (Synea 500 Air TK-100 L, CA 1:5 L Standard, StarDental 430 Torque DentalEZ) were measured, and their average was the reference value (weight: 59 g, circumference of the head: 35.4 mm), which was designated as the top of the dynamic scale (maximum point, 2.5). The bottom of the scale (0 points) represented the IOS that had the worst data (heaviest and of the greatest circumference).

iii) Scoring based on the in vitro measurements, which had two parts (maximum of 15 points):
  1. *Scoring of scanning time and continuity of scanning (2.5* + *2.5* = *maximum of 5 points) based on the 30 digital impressions*

The dynamic scoring system was set up to evaluate scanning speed as follows: throughout the procedure, the IOS observed with the shortest average scanning time represented the top of the scale (maximum score, 2.5 points), while the IOS with the slowest average operating speed represented the bottom of the scale (0 points). Regarding the continuity of scanning, the IOS with no interruption during the scanning process earned the maximum score (2.5 points), while the scanner with the greatest number of interruptions during data capture was rated at the bottom of the scale (0 points).

c. 2) Scoring of accuracy based on 15 randomly chosen digital impressions (trueness and precision; maximum of 10 points)

The average of the 15 in vitro measurements was used to calculate the score for trueness and precision; these points were then summed to indicate the IOS accuracy. We measured five different parameters, which are listed above in the Methods section. Each IOS can have a maximum of 10 points for accuracy: 5 for maximum trueness and 5 for maximum precision. The method of accuracy scoring is detailed above in the Methods section.

## Results

**Fig. 5:**
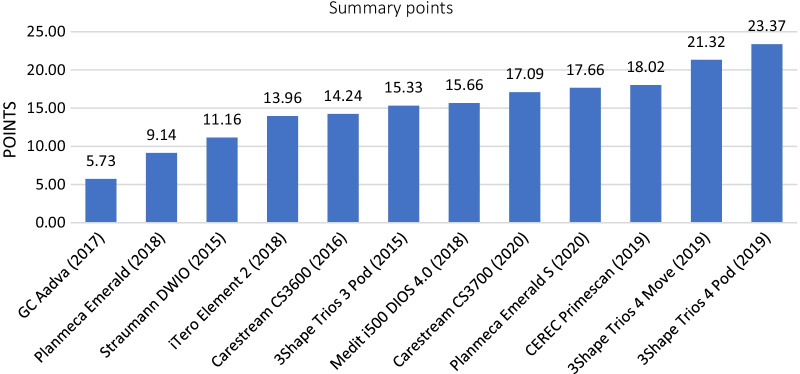
Summary points based on our measurements

Based on our measurements and tables, the 3Shape Trios 4 Pod achieved the greatest cumulative score (23.37 points). The GC Aadva scored 5.73 points out of a maximum of 30 points, which was the poorest result in our study. The other IOSs in decreasing order of their point scores were the 3Shape Trios 4 Move (21.32 points), CEREC Primescan (18.02 points), Planmeca Emerald S (17.66 points), Carestream CS3700 (17.09 points), Medit i500 (15.66 points), 3Shape Trios 3 Pod (15.33 points), Carestream CS3600 (14.24 points), iTero Element 2 (13.96 points), Straumann DWIO (11.16 points), and Planmeca Emerald (9.14 points). (Fig. 5).

**Fig. 6:**
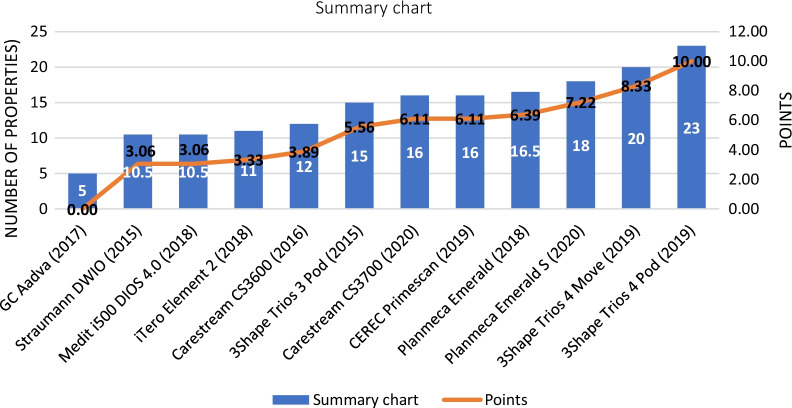
Results based on the summary chart

Looking at the results of the summary chart, the best IOS based on properties was the 3Shape Trios 4 Pod (23 properties from 26, 10 points); the worst performing IOS was the GC Aadva (5 properties from 26, 0 points). The other IOSs in decreasing order of their point scores were the 3Shape Trios 4 Move (8.33 points), Planmeca Emerald S (7.22 points), Planmeca Emerald (6.39 points), CEREC Primescan (6.11 points), Carestream CS3700 (6.11 points), 3Shape Trios 3 Pod (5.56 points), Carestream CS3600 (3.89 point), iTero Element 2 (3.33 point), Medit i500 (3.06 points), and Straumann DWIO (3.06 points). The results of the summary chart are shown in Fig. 6.

**Fig. 7:**
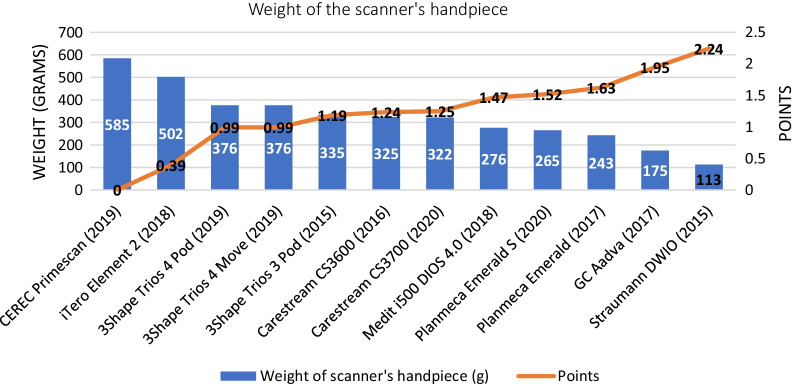
Results of the comparative assessment (weight of the scanner’s handpiece)

**Fig. 8:**
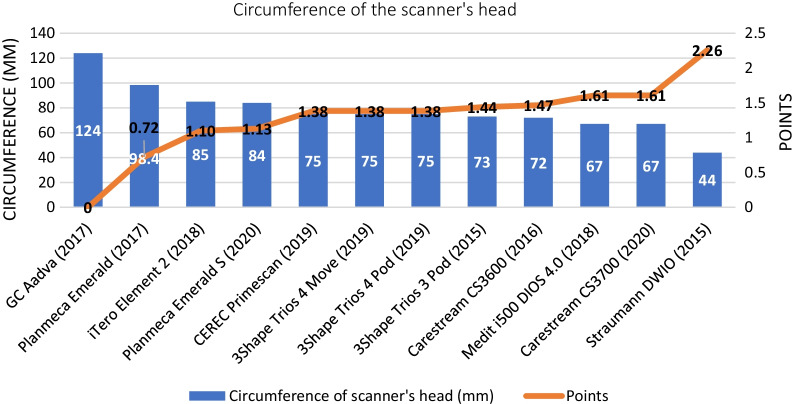
Results of the comparative assessment (circumference of the its head)

In the comparative assessment, the lightest IOS device was the Straumann DWIO IOS (113 g, 2.24 points), and the heaviest device was the CEREC Primescan (585 g, 0 points). The other IOSs in decreasing score order for weight were the GC Aadva (1.95 points), Planmeca Emerald (1.63 points), Planmeca Emerald S (1.52 points), Medit i500 (1.47 points), Carestream CS3700 (1.25 points), Carestream CS3600 (1.24 points), 3Shape Trios 3 Pod (1.19 point), 3Shape Trios 4 Move (1.00 points), 3Shape Trios 4 Pod (0.99 points), and iTero Element 2 (0.39 points) (Fig. 7). The Straumann DWIO had the smallest head (44 mm, 2.26 points), and the GC Aadva had the largest head (124 mm, 0 points). Other IOSs in decreasing order of scores for head size were the Carestream CS3700 (1.61 points), Medit i500 (1.57 points), 3Shape Trios 3 Pod (1.50 points), Carestream CS3600 (1.47 points), 3Shape Trios 4 Pod (1.44 points), 3Shape Trios 4 Move (1.38 points), CEREC Primescan (1.38 points), Planmeca Emerald S (1.13 points), iTero Element 2 (1.10 points), and Planmeca Emerald (0.72 points). (Fig. 8).

**Fig. 9:**
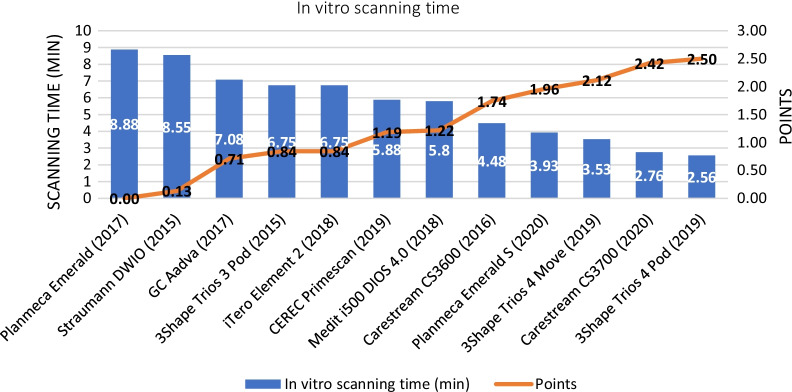
Results of the average in vitro scanning time

The results of the in vitro scanning time of our measurements are shown in Fig. 9. The fastest IOS was the 3Shape Trios 4 Pod (2.56 min, 2.5 points), while the Planmeca Emerald (8.88 min, 0 points) was the slowest device in our study. Other IOSs in decreasing order of their scores for scan time were the Carestream CS3700 (2.42 points), 3Shape Trios 4 Move (2.12 points), Planmeca Emerald S (1.96 points), Carestream CS3600 (1.74 points), Medit i500 (1.22 points), CEREC Primescan (1.19 points), iTero Element 2 (0.84 points), 3Shape Trios 3 Pod (0.84 points), GC Aadva (0.71 points), and Straumann DWIO (0.13 points).

**Fig. 10:**
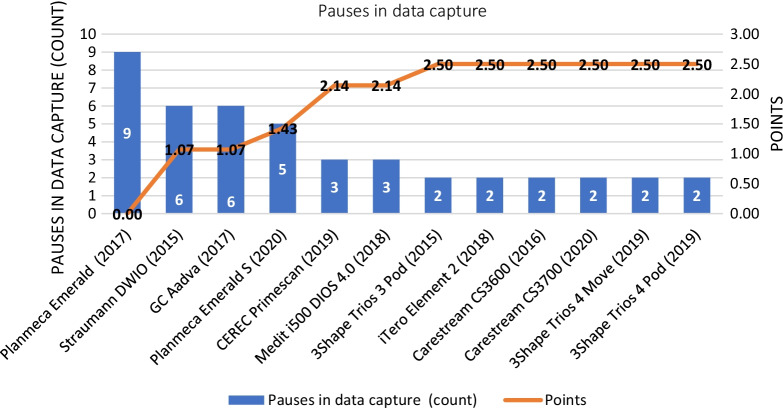
Results based on scanning continuity

Regarding scanning continuity, the best-performing IOSs, which tied at identical point scores, were the 3Shape Trios 3 Pod, iTero Element 2, Carestream CS3600, Carestream CS3700, 3Shape Trios 4 Move and 3Shape Trios 4 Pod (2 interruptions on average, 2.50 points). The worst performing IOS was the Planmeca Emerald (9 interruptions on average, 0 points). The rest of the IOSs in increasing order of their scores for scanning continuity score were as follows: the Straumann DWIO (1.07 points), GC Aadva (1.07 points), Planmeca Emerald S (1.43 points), CEREC Primescan (2.14 points), and Medit i500 (2.14 points). The results of scanning continuity are shown in Fig. 10.

**Fig. 11:**
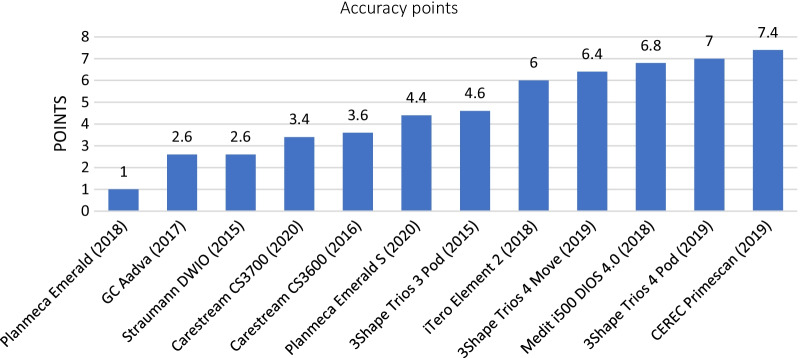
Results based on accuracy (trueness + precision)

The results of accuracy (trueness + precision) are shown in Fig. 11. The most accurate IOS in our study was the CEREC Primescan (trueness 4.2 points + precision 3.2 points = 7.4 points out of 10). Planmeca Emerald had the lowest accuracy points in our study (trueness 0.4 points + precision 0.6 points = 1.0 points out of 10). The other IOSs ranked in decreasing order in terms of accuracy scores were the Trios 4 Pod (7 points), Medit i500 (6.8 points), 3Shape Trios 4 Move (6.4 points), iTero Element 2 (6 points), 3Shape Trios 3 Pod (4.6 points), Planmeca Emerald S (4.4 points), Carestream CS3600 (3.6 points), Carestream CS3700 (3.4 points), GC Aadva (2.6 points), and Straumann DWIO (2.6 points).

**Fig. 12:**
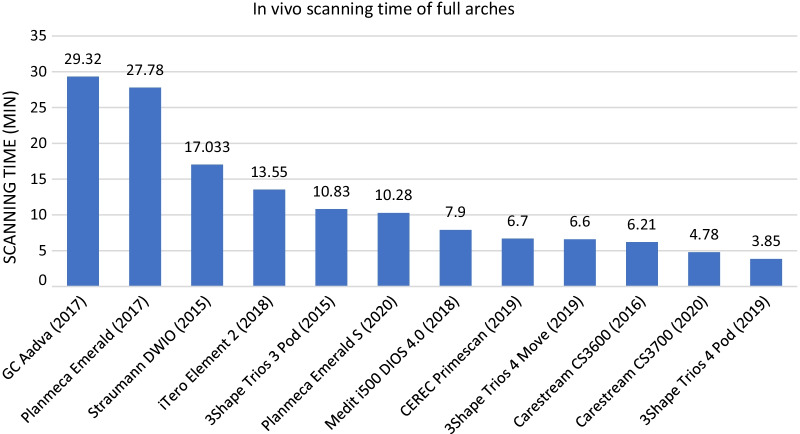
Results of the average in vivo scanning time for full arches

**Fig. 13:**
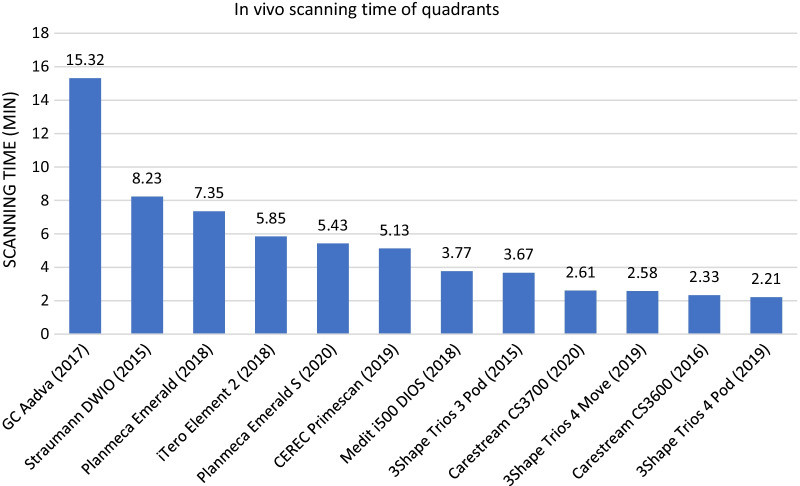
Results of the average in vivo scanning time for quadrants\

Based on our measurements, the fastest IOS for in vivo full-arch scans and quadrant scans was the 3Shape Trios 4 Pod (3.85 min for full arch, 2.21 min for quadrant). The slowest IOS was the GC Aadva for full-arch and quadrant impressions (29.32 min for full arch, 15.32 min for quadrant). The results of the average scanning time for full arch and quadrant impressions are shown in Figs. 12 and 13. Points were not awarded based on the in vivo scanning time results.

## Discussion

The introduction of dental IOSs has elicited varied feedback from dentists: at first, mostly skeptical attitudes emerged toward the accuracy of the impressions, as well as negative feelings due to prohibitive initial costs. The rejection of the paradigm shift in dental practice to changing from analog to digital dentistry is still common among dental clinicians [13]. However, there are many scientific studies in the literature on the accuracy of IOSs [5, 9, 20, 21, 28, 29, 32–40]. Most of these studies the compared digital intraoral scanning workflow to the indirect CAD/CAM workflow [11, 30, 41–43] or IOSs to conventional impression-taking methods [4, 34, 36, 44–46]. Notably, IOSs are as accurate as conventional methods or laboratory scanner devices, and they can be used for impression-taking during real prosthetic workflows [47–52]. Based on the literature, there are differences in the accuracy of IOS devices for full-arch impressions [35, 53]. In the literature, the clinically acceptable range is 50–120 µm for the marginal fit of a restoration. Above 200 µm, the restoration is not acceptable. With digital techniques, at least 50 µm is a minimum required for trueness and 10 µm for precision (11). We have less information about the validation procedures of IOSs. There is no scientific publication based on a uniform rating system; however, it is important to compare these devices in objective parameters. Before investing in a new IOS, information on IOSs available in the market is needed. Additionally, dentists need information on the accuracy, ergonomic design, scanning speed, utility of the software, and other users’ subjective opinions of the IOS. It is important to highlight the role of the distributor company because if there is any problem with the device or difficulty during the early learning curve, the members of the dental team need to be able to receive help from the distributor. Interestingly, most IOSs have their own indication area, and it is essential to use each IOS for its main indication area to achieve the perfect outcome [3]. If a dentist buys an IOS for prosthetic work, it is important to know the accuracy and features of the IOS (e.g., digital shade measurement and smile design) [50, 52, 54–61]. The first IOS capable of digital shade measurement was the 3Shape Trios 3 which was released in 2015. Now, the Planmeca Emerald S and the Carestream CS3700 are able to measure tooth shade as well [54, 62, 63]. Another important aspect is the prosthetic indication of the IOS, e.g., whether the IOS can take whole-arch impressions or the manufacturer recommends it for acquiring quadrant digital scans [40, 64, 65]. For orthodontic use, the accuracy of the full-arch study impressions is highly important as these impressions can be used to subsequently create an accurate superimposition of the cone-beam computed tomography (CBCT) scan and digital impressions [24, 66–68]. Digital technology plays an important role for dental surgeons in precisely planning the implant position during guided implant surgery [61, 69–71]. Many systems include chairside design software for prosthetic workflows. The first chairside CAD/CAM system was the CEREC; however, 3Shape and Planmeca systems also have their own design programs for chairside prosthetic workflows (e.g., inlays, onlays, crowns, short bridges) [59, 72, 73]. Our rating system is a uniform ranking scheme for all IOSs, but it is important to separately consider the findings of each assessment domain in our study. In our summary chart, the best IOS was the 3Shape Trios 4 Pod (10 points); the worst performing IOS was the GC Aadva (0 points). The GC Aadva has been available from 2017, while the Trios 4 Pod was introduced to the market in 2019. The new generations of IOSs have more specialized properties (e.g., tooth shade selection, smile design, caries detection, denture workflow, and individual jaw movement detection); therefore, their points of the summary chart were higher. This tendency can be observed between the latest and previous versions of Trios (Trios 3 Pod: 5.56 points, Trios 4 Move: 8.33 points, and Trios 4 Pod: 10 points), Planmeca (Emerald: 6.39 points, Emerald S: 7.22 points) and Carestream (CS3600: 3.89 points, CS3700: 6,11 points) IOSs. Furthermore, intraoral scanning devices differ widely in size. Related parameters (weight and circumference) are important for the convenience of the scanning process for both the dentist and the patient. In this study, the lightest and smallest IOS was the Straumann DWIO, which is no longer available in the dental market [37]. The company has a new IOS, the Straumann Virtuo Vivo. According to the manufacturer’s information, the Virtuo Vivo has better features than the previous version [74]. The heaviest IOS in our study was the CEREC Primescan because it captures more data than the previous versions of CEREC IOSs, and postprocessing starts in the IOS handpiece. Because of the operation method of this IOS, the model is considerably clear before trimming, and there is no limit to the number of digital images that can be acquired [18]. CEREC Primescan earned the lowest points of the comparative assessment (weight of IOS handpiece); however, based on the accuracy measurements, Primescan was the most accurate intraoral scanning device in our study. Pertinently, the configuration of the IOSs could also influence the ergonomic design. The Trios 3 Pod was lighter than the other Trios IOSs (Trios 4 Pod and Move) because it is a wired device. The Trios 4 Pod and Move were wireless versions and worked with the battery, which added extra weight. Regarding the practical application of IOSs, one of the most important factors is the scanning process time. In vitro and in vivo impression-taking procedures were also performed in our study. In vivo scanning times were not included in the scoring system because the standardization of in vivo measurements is unreliable; accordingly, the results of in vitro measurements were converted into points. The two fastest IOSs in our study were the Trios 4 Pod (2.56 min) and CS3700 (2.76 min), and the difference between them was irrelevant (0.20 min) in terms of practical application. Both are new generation IOSs and were available on the market from 2019 (Trios) and 2020 (CS3700). The average scanning times of Emerald S and Trios 4 Move were under 4 min, and the difference between them was not remarkable (0.40 min). Students could make digital impressions in 3.53 min with Trios 4 Move (available since 2019) and in 3.93 min using Emerald S (available from 2020). The slowest IOSs were the Planmeca Emerald (8.88 min), which is an earlier generation of the Planmeca IOSs, and the Straumann DWIO (8.55 min), which is no longer available on the market.

The continuity of scanning could influence the scanning speed and the users’ experiences. There was correspondence between the results of scanning time and stops of continuity. In the case of scanning speed, the fastest IOSs were the Trios 4 Pod, Trios 4 Move, CS3600 and CS3700, and these IOSs also had the highest points in continuity (2 interruptions on average). Continuity depends on the capacity of the computer/laptop that was used with the IOS devices. In most cases, the manufacturer determines the minimum system requirements, and following the recommendation in the interest of long-term clinical application is important. In our study, many of the examined devices were equipped with a built-in computer (cart version) or were connected to laptops provided by the distributor company.

For all the indication areas (prosthetic workflow, orthodontic or implant planning, impression machine), accuracy is a highly important parameter. An accurate STL file is crucial regarding the digital workflow and is the foundation of long-term success in digital dentistry. Based on our measurements, the most accurate IOS was the CEREC Primescan followed by Trios 4 Pod, Medit i500, 3Shape Trios 4 Move and iTero Element 2. The Primescan and the Trios IOSs are new generation devices of their company, and both have been available since 2019. The Medit i500 and iTero Element 2 were introduced to the market in 2018, and new generations were published since then. Based on the summary results of accuracy, the CEREC Primescan was the most accurate device in our study, but there were differences among the values of the examined parameters (full arch WHL, single abutment WHL, inlay WHL, and bridge and arch caliper) (Table 2). Regarding full-arch scans and arch calipers, the Trios 4 Pod had the highest accuracy (38.2 µm for trueness and 17.5 µm for precision for full arch, 74.3 µm for trueness and 74.3 µm for precision for arch caliper); however, in other parameters (single abutment, inlay cavity, bridge caliper), the Primescan accuracy results were superior to those of the Trios 4 Pod. The information of differences in accuracy among the examined parameters could be helpful for clinicians: for chairside application, the single abutment WHL, inlay WHL and bridge caliper are the relevant parameters. For orthodontic applications or laboratory-supported digital workflows, full-arch WHL is more important than other parameters. In the latter case, the digital impressions of implants are also essential, but in our study, we did not examine this parameter. The Planmeca Emerald had the lowest score for accuracy (1 point from 10), but improvement could be observed because the new generation of the Planmeca IOS (Emerald S) had a better accuracy score (4.4 points from 10) than that of the previous generation of the device. Our accuracy results were similar to the results of other studies [75–77]. Our results and points confirmed the statement that the latest generations of IOSs have improved properties over the previous versions. The Emerald S had more points in every parameter than the previous version of the IOS except for weight because the Emerald S (265 g) is a heavier IOS device than the Emerald (243 g). The Planmeca Emerald IOS scored 9.14 points; the newest version of this IOS (Planmeca Emerald S) had 17.66 points, which is 8.32 points higher. In the case of the Planmeca IOS, the primary reason for its high score points was its accuracy. The Planmeca Emerald S was more accurate in our measurements (Planmeca Emerald at 1.0 points was the most inaccurate IOS in our study, while the Planmeca Emerald S scored 4.4 points out of a maximum of 10 points). The other main outcomes were for in vitro scanning, i.e., scanning time and interruptions of data capture. The Planmeca Emerald had 9 pauses during scanning, and the Emerald S had 5 pauses. In addition, there was a considerable difference in time. The in vitro scanning time with the Emerald was 8.88 min (0 points) and 3.93 min (1.96 points out of 2.5) with the Emerald S. With the Carestream IOSs, the same tendency could be seen. The CS3600 had 14.24 points, while the new version, CS3700, had 17.09 points, the difference being almost 3 points. The difference originated from the summary chart: the CS3700 had two more features than the CS3600 (2 properties and 2.22 points in difference between the devices). On the other hand, the CS3700 was faster than the previous version: students were able to take digital impressions with the CS3600 in 4.48 min (1.74 points), while for the CS3700, the figure was 2.76 min (2.42 points). There was an interesting difference in the accuracy points in the case of Carestream IOSs. The CS3600 had more points for accuracy (2.4 points for trueness + 1.2 points for precision = 3.6 points) than the CS3700 (2.2 points for trueness + 1.2 points for precision = 3.4 points). The value of the precision was the same for each IOS, but the trueness of CS3700 was lower. In the case of the Trios IOSs, not only the different generations of IOSs but also the software versions could be examined. The oldest software version was for the Trios 3 Pod (18.1.2.), the Trios 4 Move worked with the 19.2.2. software version, which has been available since 2019, and the newest software was used in the Trios 4 Pod (20.1.1 available from 2020). The earned points also reflected the differences among the IOS generations and the software versions. Trios 4 Pod had 23.37 points out of 30, while Trios 4 Move scored 21.32 points, and Trios 3 Pod scored 15.33 points. Trios 4 had more special functions than the previous version of the Trios IOS. The Trios 3 Pod had 15 features out of 26, which translated to 5.56 points; the Trios 4 Move had 20 features (8.33 points), and the Trios 4 Pod was the best IOS in our summary chart, with 23 features scoring the maximum possible 10 points. There were also large differences in scanning time: the average in vitro scanning time with the Trios 3 Pod was 6.75 min (0.84 points); with the Trios 4 Move, the average in vitro scanning time was 3 and a half minutes (2.12 points), and the Trios 4 Pod was the fastest IOS in our study with a scan time that was one minute faster than that of the Trios 4 Move (2.56 min). There was a difference in accuracy: the Trios 4 Pod had 7 points, while the Trios 4 Move had 6.4 points for accuracy, and the Trios 3 Pod received 4.6 points.

Digital dental processes transform all workflows of dentistry and require different skills not only from dentists but also from dental technicians. It is important to highlight that IOSs should be tested in use before purchasing them. There are some limitations of this study. During the testing procedures, computers were provided by the distributor company so that they could affect the scanning procedures (e.g., scanning speed or continuity of scanning). Another limitation was the low number of digital impressions. We had a total of 10 digital scans taken by each IOS, and accuracy measurements were made based on these data. Furthermore, the ability of the dental students who made digital impressions could also influence the results.

## Conclusions

Within the limitations of the present study, the IOS devices were evaluated fairly with our uniform rating system based on a defined set of aspects (same objective parameters). The scoring system reflects the differences among IOS devices based on the evaluated objective parameters and helps Hungarian clinicians select the right IOS device for the individual needs of their dental practices. The rating system allows uniform ranking for all IOSs, but it is important to consider the results of each rating subscale (e.g., scanning time, accuracy of digital impressions, special properties). The properties of IOSs determine their main indication area, and knowledge of the differences among IOSs could be helpful for practitioners to choose the ideal device. The selection of IOSs is determined by the indication area (impression machine, orthodontic or implant planning, chairside or labside prosthetic workflow).

The new generations of IOSs have better properties than the previous versions. The new version of the devices have more specialized features (e.g., tooth shade measurement, smile design, caries detection), and their accuracy is higher than that of the previous versions. Moreover, the software updates positively influenced the examined parameters (summary chart, scanning time, continuity, and accuracy) of the scanning devices. The differences in IOS devices will decrease as new generations of IOSs and new versions of software appear on the market.

## Data Availability

The datasets generated and/or analyzed during the current study are not publicly available due the large amount of the datasets but are available from the corresponding author on reasonable request.

## Abbreviations

CAD/CAM: Computer-aided design/computer-aided manufacturing
IOS: IOS
